# Adult childhood cancer survivors’ narratives of managing their health: the unexpected and the unresolved

**DOI:** 10.1007/s11764-016-0517-8

**Published:** 2016-01-30

**Authors:** A. Fuchsia Howard, Karen Goddard, Jason Tan de Bibiana, Sheila Pritchard, Robert Olson, Arminee Kazanjian

**Affiliations:** School of Nursing, The University of British Columbia, T201–2211 Wesbrook Mall, Vancouver, BC V6T 2B5 Canada; Department of Radiation Oncology, BC Cancer Agency, Vancouver, BC Canada; Centre for Research on Inner City Health, St. Michael’s Hospital, Toronto, ON Canada; Division of Hematology/Oncology, BC Children’s Hospital, Vancouver, BC Canada; Department of Surgery, The University of British Columbia, Vancouver, BC Canada; School of Population and Public Health, The University of British Columbia, Vancouver, BC Canada

**Keywords:** Childhood cancer survivor, Health services, Patient perspective, Qualitative, Narrative research

## Abstract

**Purpose:**

Currently, 80 % of children diagnosed with cancer will be cured. However, many of these survivors go on to develop long-term health problems or late effects related to their previous cancer and therapy and require varying degrees of lifelong follow-up care. The purpose of this study was to identify the different ways that adult survivors of childhood cancer manage their medical and psychological challenges.

**Methods:**

Data from in-depth interviews with 30 adult survivors of a childhood cancer (9 to 38 years after diagnosis, currently 22 to 43 years of age, 60 % women) were analyzed using qualitative, thematic narrative analysis methods.

**Results:**

The survivors had not expected the medical, psychological, and social challenges that arose over time and that often remained unresolved. Five narrative themes revealed distinct ways that survivors managed their health challenges: (1) trying to forget cancer, (2) trusting the system to manage my follow-up care, (3) being proactive about my health, (4) stumbling from one problem to the next, and (5) struggling to find my way.

**Conclusions:**

Variation exists in the ways in which childhood cancer survivors frame their health, their perceived significance of health challenges, strategies used to manage health, interactions with healthcare professionals and the health system, and parental involvement.

**Implications for Cancer Survivors:**

This research provides novel insights that can be used to inform the development of patient-centered health services that promote the assessment and tailoring of care to the diverse ways survivors enact their agency, as well as their psychoeducational coping styles, therapeutic relationship needs, and information needs.

## Background

Childhood cancer cure rates now exceed 80 %, leading to evergrowing numbers of cancer survivors living into adulthood [1, 2]. Unfortunately, many of these childhood cancer survivors (CCS) will face an increased risk for a broad range of serious physical and psychological health conditions during adulthood [3–7]. The prevalence of late effects is considerably higher than originally anticipated and disclosed. In the St. Jude Lifetime Cohort Study [4], by age 45, the estimated cumulative prevalence of any chronic health condition was 95 % and a disabling life-threatening chronic condition was 80 %. The most common adverse health outcomes in this research were pulmonary dysfunction (65 %), hearing loss (62 %), endocrine/reproductive dysfunction (62 %), cardiovascular disease (56 %), and neurocognitive impairment (48 %) [4]. Psychological late effects are also profoundly disabling, particularly anxiety, depression, fear, and posttraumatic stress [8–10]. There is considerable variability in late effect risks. Possibly one third of CCS will experience relatively few minor complications and face minimal long-term risks, yet the full effects of treatment on the aging of different organ systems will only become evident as the population of CCS ages [11]. There is strong evidence that the majority of serious health problems do not become apparent until many years, even decades, after the cancer has been cured, and these late effect risks increase substantially with age [3, 5].

There is general consensus, endorsed by leading North American and European organizations, that CCS require risk-stratified, lifelong follow-up care that includes screening for recurrence and secondary cancer, and surveillance and treatment of physical and psychosocial late effects [12–15], based on the previous cancer, cancer therapy, genetic predisposition, lifestyle behaviors, and co-morbid health conditions [11]. Yet, a significant proportion of adult CCS is not receiving this screening [16, 17], in part, because a general lack of awareness of late effects by survivors, a lack of capacity for survivor care in cancer institutions, physician unfamiliarity with the health needs of survivors, and a lack of communication between survivors, cancer centers, and primary care physicians [18–28].

The development, implementation, and refinement of long-term follow-up (LTFU) services remain an ongoing effort in countries worldwide. There have been initiatives to establish formal LTFU programs, link survivors with medical providers, educate primary and specialist healthcare providers, develop and disseminate evidence-based practice guidelines [29], share medical information among medical providers, empower CCS with information, and encourage survivor involvement in their care [30]. Indeed, the facilitation of self-management among cancer survivors in general [31], and survivors of childhood cancer in particular, is increasingly recognized as essential [22, 32]. Self-management support is now a priority in Canada and is supported by evidence that chronically ill individuals who are engaged in self-management have reduced disease-related effects and more effective use of health services [33]. Despite LTFU initiatives and the recognition of the potential benefits of CCS self-management, there remains a need to explicate and integrate the perspectives of CCS into such laudable efforts. Survivors’ perspectives have remained on the periphery of health service development and policy making [34–36] despite being foundational to patient-centered services.

The limited patient-perspective research describes adult CCS’s feeling as though cancer never ended; frustrations with ongoing consequences of treatment; their functional status; unsatisfactory dialogue with healthcare professionals (HCPs); difficulty separating cancer from their self-identity; and emotions related to uncertainty, fear, trepidation, and anxiety for their future [37–41]. Tsonis and colleagues [39] described strategies adult CCS used to improve their quality of life and cope with lasting impacts of cancer including adopting a positive outlook, living a healthy lifestyle, and seeking out others, the use of which were mediated by personal and environmental resources of knowledge, relationships, community, technology, and medications. The next step is to detail survivors’ perspectives and experiences of how they manage new and chronic physical and psychological challenges and their risk of late effects. Accordingly, the purpose of this research was to provide an in-depth description of the different ways that adult CCS manage their medical and psychological challenges.

## Methods

We used a qualitative, thematic narrative analysis approach [42] to guide this research. An underlying assumption of all narrative analyses approaches is that individuals make sense of past events and actions they deem important in their lives, especially difficult life transitions and illness, by telling stories that link events or ideas sequentially [42, 43].

### Guiding analytic theory

The theory of relational autonomy guided the collection and analysis of data [44, 45]. Relational autonomy highlights the interconnectedness and interdependence of individuals, and the dynamic balance among people who are closely involved in each other’s lives [46]. A relational view of autonomy characterizes the individual as embedded in relational networks that include family, friends, and HCPs, as well as institutional, political, and social systems [44, 47, 48]. These relational networks are accompanied by social obligations, including roles and responsibilities, which provide the framework within which individuals act [44]. A relational autonomy lens was initially used to formulate the interview guide to enable an exploration of adult CCS experiences in the context of their interpersonal relationships. Participants were asked to reflect on how family, friends, HCPs, and organizations helped and/or hindered them in managing or coping with physical and emotional health issues, and during data analysis, particular attention was paid to the participants’ descriptions of the different roles of family members, peers, and HCPs and how these changed over time.

### Study setting and participants

This research was conducted in the province of British Columbia, Canada, where there is a publicly funded healthcare system. Study participants who were diagnosed with cancer prior to 19 years of age and between the ages of 19 and 45 years at the time of study were recruited. Study fliers were distributed to potential participants who attended a post-pediatric late effect clinic at the adult cancer center and a LTFU clinic at a children’s hospital. Study notices were also posted in relevant online forums and websites.

All eligible survivors who contacted the research team were interviewed, and purposive sampling was used to further recruit participants with diverse characteristics (age at time of diagnosis, current age, diagnosis, and rural and urban areas of residence) and experiences until data saturation was achieved. A total of 30 long-term CCS participated, of which 25 were recruited through a clinic and five were recruited through an online forum or website for cancer survivors. Participants ranged in age from 22 to 43 years (mean 31 years) at the time of interview and were 9 to 38 years (mean 22 years) from the time of diagnosis. See Table 1 for demographic information and Table 2 for disease characteristics and late effects.

**Table 1:** Participant demographic information, by dominant narrative theme

Demographic characteristics		All, *n* = 30	Trying to forget, *n* = 3	Trusting the system, *n* = 9	Being proactive about my health, *n* = 11	Stumbling from one problem to the next, *n* = 5	Struggling to find my way *n* = 2
Age	20–24	5	1	3	1	0	0
	25–29	8	1	2	3	1	1
	30–34	9	0	1	4	3	1
	35+	8	1	3	3	1	0
Gender	Male	12	3	2	4	3	0
	Female	18	0	7	7	2	2
Place of residency	Greater Vancouver area	21	2	6	6	5	2
	Other	9	1	3	5	0	0
Marital status	Single	22	3	8	6	3	2
	Married	8	0	1	5	2	0
Living arrangement	Alone	9	1	3	3	1	1
	With roommates	4	0	1	2	1	0
	With a partner/spouse	8	0	1	5	2	0
	With parents	9	2	4	1	1	1
Level of education	Did not complete high school	2	1	1	0	0	0
	Completed high school	7	1	3	0	1	2
	Completed university/college	21	1	5	11	4	0
Employment status	Unemployed	4	1	2	1	0	0
	Student	3	0	0	0	2	1
	Employed part- or full-time	23	2	7	10	3	1

**Table 2:** Participant disease characteristics and late effects, by dominant narrative theme

Disease characteristics and late effects		All, *n* = 30	Trying to forget, *n* = 3	Trusting the system, *n* = 9	Being proactive about my health, *n* = 11	Stumbling from one problem to the next, *n* = 5	Struggling to find my way, *n* = 2
Age at first diagnosis	0–4	8	1	4	2	1	0
	5–9	10	1	3	3	2	1
	10+	12	1	2	6	2	1
Type of cancer	Leukemia and lymphoma	16	1	6	7	2	0
	Brain tumor	6	1	0	1	2	2
	Sarcoma (not including the brain)	6	1	1	3	1	0
	Other solid tumors	2	0	2	0	0	0
Treatments	Radiation therapy	27	3	8	9	5	2
	Chemotherapy	29	3	9	11	5	2
	Surgery	11	1	2	3	3	2
	Bone marrow transplant	1	0	1	0	0	0
Late effects and health problems	Anxiety or depression	14	2	4	3	3	2
	Impaired growth and development	13	1	4	4	2	2
	Bone, joint, or soft tissue late effects	12	1	4	6	1	0
	Second cancer	9	0	2	4	1	2
	Learning difficulties or cognitive impairment	9	1	1	4	1	2
	Impaired sexual development or infertility	9	0	2	4	3	0
	Endocrine late effects	9	1	2	5	1	1
	Hearing impairment	8	1	0	3	3	1
	Visual impairment	7	0	3	1	2	1
	Digestive late effects	6	0	3	2	0	1
	Respiratory late effects	5	0	3	2	0	0
	Cardiovascular late effects	5	1	2	1	1	0
	Dental late effects	4	2	0	2	0	0

### Data collection

One investigator (FH) conducted 30 in-depth interviews lasting 45 to 120 min. Twenty of the interviews were in person, while 10 were conducted over the phone to accommodate participant availability and place of residence. All interviews were digitally recorded and transcribed verbatim. A semi-structured interview guide with open-ended questions and probes was used to ensure common themes and topics were explored with all participants, and participants had the opportunity to share their perspectives and narratives. For example, the survivors were asked to describe how their health and wellbeing changed over time; how they managed their previous and current medical and psychosocial challenges; who they talked to about their medical and psychosocial challenges; how individuals (family, friends, and HCPs) and organizations (healthcare facilities, support societies, volunteer groups) were a help or hindrance; and instances when health service needs were met and unmet. At completion of all interviews, field notes were recorded noting survivors’ responses to the questions, and social and contextual factors that might have influenced the interview process.

### Data analysis

Compared to other narrative approaches, the emphasis of a thematic approach is the content of participants’ stories more so than how a narrative is spoken, structures of speech chosen, the audience, or the local context [42]. This approach aims to uncover and categorize thematically individuals’ experiences of health and illness by keeping participants’ stories intact and theorizing from the case rather than component themes across cases [42]. Each participant’s transcript was read numerous times in an attempt to consider the transcript as a whole, as opposed to initially breaking individual transcripts apart for themes. Consistent with the process of re-storying [43], constructed narratives were then developed for each participant that summarized the forms of storytelling, the self-identity portrayed, their cancer history, medical and psychosocial challenges, significant events and experiences related to these challenges, the role that individuals and organization had played, and how survivors framed their future. Data related to social isolation, social life, and social support was extracted from the transcripts and narratives, analyzed separately, and published elsewhere [49]. The constructed narratives for all 30 survivors were compared and contrasted to identify the main narrative themes, which can be considered a typology of how adult CCS manage their medical and psychological challenges. For each survivor, a main narrative was identified based on the degree to which their forms of storytelling and experiences were represented by that narrative theme, while a theme was considered minor if it occurred less frequently or to a lesser degree. A summary was then constructed for each of the main narrative themes by drawing on the stories told by the survivors and reflecting their words.

### Findings

The framing of health issues and late effect risks as unexpected and unresolved was a primary theme woven through each survivor’s interview. Five main narratives were identified that captured the ways the adult CCS managed their medical and psychological challenges, including trying to forget cancer, trusting the system to manage my follow-up care, being proactive about my health, stumbling from one problem to the next, and struggling to find my way (see Table 3 for a summary of the key components of the narratives). Each survivor not only used one of these narratives as their main narrative but also used one or two of these narratives as minor narratives throughout their interview. Moreover, the narratives shared by the survivors were not always independent, and often were combined or interrupted by other narratives. When a survivor used more than two narratives, which were sometimes complimentary and other times contrasting, this often coincided with different times in their lives and throughout their survivor trajectory.

**Table 3:** Key components of narrative themes

	Framing of health	Self-identity portrayed	Significance of health challenges	Strategies used to manage health	Interactions with healthcare professionals	Parental involvement	Framing of the future
Trying to forget	Healthy	Just like peers Cancer in the past	Downplay issues Medical issues constant sources of anxiety and depression	Avoid medical care Substance abuse and gaming	Avoid healthcare professionals	Parents not involved/survivors disengage parents	Focus on living a normal life that would 1 day include career and family
Trusting the system	Generally healthy, but with exceptions	Cancer taught them to appreciate life and put obstacles into perspective More mature than peers	Acknowledge medical and emotional issues Reassured by medical care	Rely on others to organize care Accept recommended screening and treatment	Listen and do not seek additional care, opinions, or information Have faith in health care professionals	Learn to be independent of parents	Focus on career, relationships, and hobbies
Being proactive about my health	Health is compromised	An experienced, knowledgeable, proactive patient	Experience multiple issues Worry and anxiety ever present	Seek out information Proactive in organization and management of care Engage in healthy lifestyle behaviors	Seek assistance from existing and new healthcare professionals Request information, investigations, and referrals	Independent of parents Limit discussions with parents to protect them from worry	Optimistic but expect health issues
Stumbling from one problem to the next	Cancer never ended	Embody cancer and refer to themselves as different than their peers	Medical challenges interfere with all aspects of daily life Emotional exhaustion related to chronic conditions Despair that late effects will continue	Trust and adhere to medical recommendations	Followed by numerous healthcare professionals Psychological support often ineffective	Feel abandoned or unsupported by parents who are no longer involved	Assume future will be truncated by health issues
Struggling to find my way	Health is a long list of late effects	Consumed by health issues	All-consuming Despair, frustration and confusion with health, disabilities, and healthcare professionals	Unable to self-manage health	Loss of close relationships with pediatric providers Negative interactions prevalent	Unable to manage independently their complex health needs Appreciated unwavering parental support but desired independence	Fear their future

### The unexpected and the unresolved

The survivors expressed shock, surprise, distress, anxiety, and worry when they developed the unexpected physical and emotional late effects stemming from their initial cancer and treatment. These reactions were amplified in the face of severe, life-threatening conditions, as expressed by a 31-year-old brain tumor survivor, “The big shock was when my heart started to grow. I think it was five years or six years ago… And that was the big scare for me… I thought I was gonna not be here.” This was especially surprising and distressing for those survivors who had experienced a time of good health, relatively few medical challenges, and limited medical follow-up.

Prior to developing late effects, some survivors were virtually unaware of their future health risks, whereas other survivors had specific knowledge of existing health issues and certain risks but were not aware of the extent and severity of other risks.

“Maybe that’s why the kidneys were such a piss off because focusing on potential recurrence of cancer or thyroid or cardio and then bang, this thing that I hadn’t anticipated… I think because I saw cancer as being a time limited thing, when the kidneys were discovered (kidney failure) (I had) a frank conversation with the oncologist of what else might come up because I didn’t want to keep on having surprises every five years.” [38-year-old, Ewing’s sarcoma survivor]

The survivors who recalled being informed of their health risks when they were younger, some at the time of treatment and others during medical follow-up, were still surprised and distressed when these became a reality. Many had struggled to interpret the probability and uncertainty of late effect risks and thus perceived their risks in absolute terms, that is they considered themselves either 0 or 100 % at risk. HCPs also interpreted their risks as absolutes, which at times reinforced the survivor’s understanding and at other times challenged their thinking.

Over time, the survivors came to understand that there was limited effective treatment for many of their physical and emotional challenges and that despite ongoing treatment, these would remain unresolved. Most of the survivors also became aware that their health risks were in fact lifelong risks. Yet, it took time for the survivors to come to fully appreciate all of their risks.

### The narrative of trying to forget cancer

“Trying to forget cancer” was the dominant narrative shared by three male CCS and was a minor narrative shared by six survivors (4 women and 2 men). This narrative can be summarized as follows:

Being a cancer survivor has no impact on anything, I just live a normal life. I have virtually no medical issues. I just forget about a lot of these things. The last thing I want to do is relive that horror. I routinely miss appointments. I avoid doctors, if I can. Those things [late effects and risks] are not worth worrying about.

The brief and coherent stories presented in this narrative were devoid of medical details and often described in the past tense, wherein survivors indicated they used this approach when they were younger. The survivors presented themselves as “healthy,” “just like anyone else,” and similar to their peers, an identity reinforced by minimizing their cancer history. The survivors downplayed their own cancer experiences by drawing attention to the deep and prolonged suffering of other CCS during treatment and afterwards. The narrative of trying to forget cancer was framed as an extension of willing oneself well, a means of effective coping during cancer treatment.

“I don’t want to be defined as the guy who was sick. When I was quite sick I fought against it. I know that’s a result of being fifteen years old and not wanting to be sick, so in those brief intervals between hospital visits you do everything in your power to act like you’re not sick. So I think that’s extended into later life.” [36-year-old, leukemia survivor]

In an attempt to “put cancer in the past,” the survivors downplayed and ignored medical issues that arose, such as tooth decay, facial hypoplasia, weight gain, and hearing loss, even when these interfered with daily life. The survivors did not want their medical issues to prevent them from living a “normal life” that would 1 day consist of a successful career and a family. Despite the survivors’ attempts to minimize and ignore medical issues, these were constant sources of anxiety and depression, for which the survivors employed specific strategies to forget. They avoided hospitals, medical clinics, medical and dental appointments, telephone calls, and discussions with any HCP, be it a family doctor, an oncologist, a dentist, or a nurse. When a parent encouraged the survivor to seek out medical or emotional care, the survivor shut down the discussion and, at times, disengaged from this relationship. The survivors also distanced themselves from other cancer survivors who were reminders of compromised health and the threat of death. Some survivors relied on playing video games, using alcohol and/or drugs, and, in one case, self-mutilation, as a means of escaping physical and emotional troubles.

“It’s kind of sad honestly. I know that when I get older something might go wrong. I look at video games and like, okay, I’m forgetting about it, I’m not paying attention to like life, I’m not, I’m not paying attention to all the depression. I mean even when I was at work I was starting to have depression, depression inside. I’ll listen to, or I’ll see, or I’ll hear, that will make me start thinking in the depression spot, but then when I’m gaming I’m concentrating on that one thing.” [24-year-old, rhabdomyosarcoma (head) survivor]

### The narrative of trusting the system to manage my follow-up care

“Trusting the system to manage my follow-up care” was the dominant narrative theme shared by nine CCS (7 women and 2 men) and a minor narrative shared by eight survivors (2 women and 6 men). This narrative can be summarized as follows:

I simply accept my medical follow-up as one of the consequences of cancer. My regular check ups are just routine. I have testing done every two or three or five years because my doctor wants me to. I get emotional every time I go, but I feel very reassured. I have faith in my oncologist and GP and I put my life in their hands and I just rely on what they say.

In these matter-of-fact and easy-to-follow stories, events were placed in chronological order; yet, details were glossed over and survivors required prompting to divulge the specific nature of their physical health issues. The survivors portrayed themselves as “generally healthy,” but with exceptions, such as being overweight, a little shorter and stiffer, tired, or more susceptible to infections. Cancer was, in part, characterized as a teacher and beneficial in that the survivors have a greater appreciation of life, they are able to put the obstacles and stresses of life into perspective, and they are more mature than their peers. Framing cancer in this manner empowered the survivors to focus on “moving on” with their lives by investing their time and energy into their social life, relationships with friends and family, career, and hobbies. However, they also did not want to appear ungrateful for surviving cancer and were surprisingly hesitant to seek out or request care.

While the survivors acknowledged their medical late effects and future health risks, they left the responsibility of managing their health to others, as they had done previously. They relied on individuals in the healthcare system to schedule and remind them of regular medical appointments and to share their medical information with all relevant HCPs. The survivors trusted that they were being “looked after” by nurses and physicians who knew their cancer and treatment history and the related health risks. They accepted, without question, recommended medical treatment, such as growth hormone therapy or reconstructive surgery, as well as screening and surveillance for health risks, such as an echocardiogram or thyroid ultrasound. The survivors only briefly mentioned their emotions of worry and anxiety, instead calling attention to the considerable reassurance garnered.

“What has been recently brought about with my thyroid and possibly like radiation to my brain, yes, that concerns me a great deal, but I also love that I’m having a test every couple of years or every four years that’s helping me to make sure that’s not there.” [39-year-old, acute lymphoblastic leukemia survivor]

Comprehending complex medical information was difficult for the survivors at times, yet they did not seek out additional information, choosing instead to place their confidence in others.

“Most of the time I get it and then there’s other times where I’m really confused, like this situation is because of this and this is because of this and am I okay and I’m confused now because it’s all jumbled together. When I’m listening to the doctors they use kind of like big words so I’m not really good with big words, I don’t know what you’re talking about but, okay, keep going, I’ll catch up in a minute… I’ll have them explain it to me a little bit and see if I can get it, which for the most part I can’t.” [24-year-old, acute lymphoblastic leukemia survivor]

Stories of the survivors learning to be independent from parents who previously managed all aspects of their health also figured prominently. The parents who encouraged reliance on healthcare professionals reinforced the narrative of trusting the system.

“She’s [mother] been kind of the one taking care of my appointments, the one that goes to my doctor’s appointments with me and now I’m kind of leaning away from her. So I’m going to my appointments by myself, telling her what days they are but she can’t make it because she’s busy working. So I kind of have to do it all myself now… She [mother] always says, I’m sorry that you’re in pain but I can’t really do anything, right? Just tell the doctor, let them know what kind of pain that you have.” [22-year-old, acute lymphoblastic leukemia survivor]

However, the survivors also encountered difficulty keeping their parents informed of their health status because of their lack of comprehensive knowledge, to whom survivors would “give vague answers.” This further reinforced the survivor’s dependence on HCPs. The two married survivors, who predominantly shared the narrative of trusting the system, made efforts to keep their spouse up-to-date of their health issues when the situation warranted it.

### The narrative of being proactive about my health

“Being proactive about my health” was the dominant narrative theme shared by 11 CCS (7 women and 4 men), and a minor narrative shared by seven survivors (4 women and 3 men). This narrative can be summarized as follows:

I have repercussions that I worry about because of cancer. At first I was lost, no-one was looking out for my health and my concerns were often dismissed. Now I’m very proactive and persistent about my health. I’m informed, I have all my documentation, I can get a second opinion, I can access resources, and I know what to keep an eye on. I’m optimistic but I don’t see my life without these small and maybe one day big health issues.

This narrative consisted of detailed, chronological stories told by survivors using medical language and conveying self-confidence in managing health-related events. CCS framed their health as compromised because of ongoing medical and psychological consequences of their cancer, just as one 25-year-old, rhabdomyosarcoma (uterus/abdomen) survivor stated, “It just means that I need to kind of look after my health in a different way just be more attentive to my health. Everything kind of revolves around my health.” Worry and anxiety related to existing and potential late effects were ever present, as was distrust that they would receive the necessary health care.

In this narrative, the survivors often recounted a time when they were lost to follow-up and did not receive screening or even treatment for long-term and new health issues.

“They [Children’s Hospital] sort of let you go and sent, sent your records off to your regular general practitioner. You don’t have anyone checking on you on a regular basis right? You’re out in the open and you’re going, okay, well gee what should I be looking for? If you do get some sort of ailment you’re kind of in the back of your mind you’re going well, should I be worried about this?” [35-year-old, acute lymphoblastic leukemia survivor]

The process of becoming reconnected with a HCP who was interested and knowledgeable about their specific health issues was difficult for many, as was learning to access and navigate adult health services. The survivors also provided numerous detailed accounts of HCPs who were naïve and dismissive of the survivors’ symptoms, as described by a 24-year-old, rhabdomyosarcoma (head) survivor, “I explained to the dentist in great detail that my dental problems are from the radiation, but he was still convinced that they were a result of eating too much sugar.”

The survivors framed these negative experiences as a personal call to action, wherein they became expert, knowledgeable advocates for themselves, which consisted of asking questions, seeking out information and resources, obtaining their medical records, ensuring HCPs had their medical information, explaining their cancer history and late effect risks, requesting medical investigations (i.e., blood tests, biopsies, and ultrasounds), seeking second opinions, requesting referrals to specialists, contacting specialists directly when they were not referred, changing HCPs, and engaging in healthy lifestyle practices (i.e., diet and exercise).

“I just feel like no-one is really looking out for my health. I feel like not only am I left on my own but I feel like you’ve got to have the due diligence to research the symptoms and whatnot beforehand, before you go to see the doctor because they’re so quick to just say, well here’s a prescription off you go. If I’m concerned about something, I always start with Google. It just gives you sort of not a better understanding but maybe, just a little bit more information so when the doctor kind of fluffs you off you can kind of say well, I heard this or I read about this, can this help me or harm me in any way?” [31-year-old, Hodgkin’s lymphoma survivor]

While the majority of the survivors felt encouraged to be proactive and involved in the management of their care, there were also stories wherein HCPs were offended, resistant, or angry.

“The doctor that she [obstetrician] shared the practice with kicked me out because he didn’t like that I was so involved in my care. I knew that the pregnancy increased my risk for cardiomyopathy. I wanted to speak to a cardiologist. Instead of allowing me to see a cardiologist he [doctor] said, oh no, you just can’t have a vaginal delivery. And I said well according to my obstetrician I can. And he says, no, you can’t have a vaginal delivery, it will put too much strain on your heart. And I said well I’d like to speak to a cardiologist and that kind of miffed him and I said maybe you should talk to Dr. X [oncologist] because she can give you more information about my background, because he wasn’t willing to listen to me. And I waited and I waited and I waited for this guy to get on it and he didn’t, so I contacted Dr. X [oncologist] myself. And she said, oh no, it’s fine, you can have a vaginal delivery. You’ve got an obstetrician there, but you should see a cardiologist. So I took this back to him and he was livid that I’d gone over his head.” [34-year-old, acute lymphoblastic leukemia survivor]

Taking over the management of their medical care from their parents and learning to be proactive was difficult at first but eventually reinforced the survivor’s sense of independence and confidence in their abilities. Recognizing the emotional toll their cancer treatment had on their parents, the survivors wanted to protect their parents from any further emotional pain. As such, the survivors limited discussions and downplayed their medical issues with their parents. Similar to others, one 29-year-old acute myelogenous leukemia survivor explained that, “She’s [mother] always, always worried that there’s something going on, right. Now I just keep her in the dark pretty much… I just don’t want to worry her.” In contrast, all five married survivors whose main narrative was being proactive about my health emphasized the tremendous support provided by their spouse. The specific form of support varied among couples, yet central to this support was a match between the type of support desired and that given by the spouse. While having children triggered significant worry that they would not be able to watch them grow up if they became fatally ill, this also served as a prime motivator for all four survivors with children to be proactive.

### Stumbling from one problem to the next

“Stumbling from one problem to the next” was the dominant narrative theme shared by five CCS (2 women and 3 men) and a minor narrative shared by four survivors (3 women and 1 man). This narrative can be summarized as follows:

Cancer has always continued. It’s tough not being able to identify what is causing these problems or what we can do to lessen the impact on my day-to-day life. Not being able to see any improvement in anything. Depression and social isolation have been constant. I don’t get any answers or support. My parents are not there for me and I need them. I don’t know whether I have another thirty years.

This narrative was overflowing with long, detailed stories of medical health issues, with an emphasis on the marked emotional and social challenges that define the survivors’ lives. One story often spiraled into the next, building on prior remarks and picking up further details as the interview progressed. The survivors attempted to portray themselves as capable, independent, and optimistic, yet they also discussed feeling vulnerable, helpless, and hopeless. Although cured of their cancer, the development of late effects and need for ongoing medical treatment lead the survivors to feel that, “It doesn’t feel like I really finished anything. It’s always, cancer has always been part of my life so it’s always just kind of continued” (38-year-old, acute lymphoblastic leukemia survivor). As a result of their continued embodiment of their cancer experience, the survivors referred to themselves as “not normal,” “different,” and “fundamentally changed.”

The numerous health issues described by the survivors were serious and debilitating, including for example, neurocognitive impairment, marked hearing and visual impairment, facial and spinal hypoplasia, hypopituitarism, and infertility. These late effects often emerged in early adulthood, a time usually defined by optimum health.

“I’m on disability for this chronic migraine that we think is related to the treatment so I get a lot of migraine headaches. This year the skin cancer, two years ago a meningioma. I’ve got a dry eye too as a result of the infection so I’m having problems with that at the moment so it’s just, I don’t know there always seems to be something going on… I think it got worse, you know, I had a period after treatment where I was quite good and then these late effects are starting to crop up now.” [34-year-old, acute lymphoblastic leukemia survivor]

A deep sense of despair that their late effects will continue was pervasive, as was emotional exhaustion related to the chronic nature of untreatable disease and dysfunction.

“They’ve [doctors] tried medications, they’ve tried exercise, we’ve tried diet, we’ve tried sleep and hygiene, we tried other stuff. Anything that they can think of… Not being able to identify what these issues are or what is causing them or what we can do to alleviate them. Or maybe not even alleviate but to lessen the impact on my day-to-day life, not being able to see any improvement in anything we’ve tried. It’s been going for almost eleven years, it’s tough.” [28-year-old, brain tumor survivor]

The survivors felt they had no choice but to trust and adhere to recommendations made by their multiple HCPs owing to the complexity of their medical issues. Yet, traveling to appointments with multiple HCPs (i.e., oncologists, endocrinologist, cardiologists, dentists, pulmonologists, and ophthalmologists) and for various medical investigations (i.e., magnetic resonance imaging, mammogram, and ultrasound) that were far from home was costly and exhausting and involved taking time away from studies or off work. The survivors also described the financial costs associated with crucial medical devices and treatment (i.e., hearing aids, growth hormone replacement therapy, and in vitro fertilization), for which some had no healthcare insurance, resulting in some forgoing treatment.

Stories of medical challenges that plagued all aspects of daily life dominated. The survivors explained how physical impairments interfered with their ability to work, study, maintain an active and healthy lifestyle, and engage in meaningful social and intimate relationships. Coping with worry, fear, anxiety, depression, and social isolation were ongoing.

“It’s hard to just plow through the shit right now, pardon my French but. I mean an analogy that I use for my energy is people walking so walking through air it’s fairly easy. I mean if you get into like water it’s very difficult to walk. And then if that water gets more dense it’s even harder to walk. So I feel like most people are just walking through air but I’m going different depths of water, different intensities of water consistently so I mean that’s an analogy I try to use for how difficult it is for me just to, to get through the day.” [28-year-old, brain tumor survivor]

While some survivors were able to access psychosocial services and support, these were often ineffective, and as such, the survivors felt demoralized and had begun to lose hope. The survivors also felt abandoned and unsupported by their parents who no longer accompanied them to appointments and communicated with HCPs on their behalf. While the parents were considered somewhat sympathetic, they no longer witness the survivor’s day-to-day challenges and, thus, did not appreciate the severity of their numerous medical, emotional, and social challenges. In turn, the survivors limited the disclosure of personal information and made minimal attempts to keep their parents up-to-date because they were “hard to talk to” and not able to provide the level of support they once did. The two married survivors who predominantly shared this narrative characterized their medical and emotional challenges as considerable sources of distress for their spouse and ultimately, marital discord.

“It [infertility testing and treatment] was very difficult to discuss, it was very difficult. And I think maybe in part because there was no lead up to it, there was, I think there was complete shock on both of our ends about it and that, that just created a, a real barrier for him [husband] that he was not able to talk about it, he was just devastated.” [32-year-old, Hodgkin’s lymphoma survivor]

### Struggling to find my way

The narrative of “struggling to find my way” was the dominant narrative theme shared by only two women CCS and the minor narrative shared by two other survivors (1 woman and 1 man). This narrative theme can be summarized as follows:

I still get double vision, my hearing is dropping, my balance is off. And I have slow motor skills, I use wrong words, I have slow thinking and a slow response. And the childhood doctors can’t help me because I reached a certain age. I hate adult doctors. My dad will go with me to the doctor but, I want to be independent. I’m an adult, before I had lots of help but now I worry about what will happen? I want to be happy but I don’t know what that is, I'm just coping with life.

In this narrative, the survivors told disorganized and difficult-to-follow stories of debilitating late effects. They were unable to see beyond a self-identity consumed by a long list of medical and psychosocial issues and presented themselves as struggling with all aspects of life. This was accompanied by pervasive depression, worry, loneliness, and hopelessness.

The survivors touched briefly on their medical challenges but primarily focused on their unsuccessful attempts and frustrations with their health, disabilities, and HCPs. The transition from pediatric to adult services represented the loss of close relationships with pediatric care providers, with whom they had long-standing emotional bonds. They felt “kicked out” of pediatric services and unsupported by adult HCPs. Moreover, the survivors shared exasperating stories featuring HCPs who lacked compassion and were condescending.

“When I see her [doctor] she said, “when was your last eye check?” And I said, “I don’t know I think ten years ago.” And then she said, “okay, let me see the copies,” and I said, “I don’t have any copies.” And so she said, “okay, well after ten years most likely doctors don’t have your, your records.” And I said, “well I don’t have any copies but my family doctor has copies.” And so she said as I left, “okay, this is what you need to do blah, blah, blah because that’s what adults do.” And then the second appointment with her once again she did another eye test and she said,” okay, now keep this record because that’s what adults do,” and that pissed me off. That she was talking to me like I was a kid. As if I was being a, that naïve on purpose.” [38-year-old, astrocytoma survivor]

Over time, the survivors felt less inclined to seek out assistance.

I used to go to the counselor but I stopped because I felt judged the last time I guess… I think he kind of raised his voice where I felt he was starting to get frustrated and then I guess that hurt my ego. Dr. [name] said I should give him a second chance. But I think I’m, I’ve had moments like this a lot that I’m starting to procrastinate about giving second chances.” [38-year-old, astrocytoma survivor]

There were no effective treatments for many of the complex medical and emotional challenges facing the survivors, resulting in the survivors constantly struggling with their chronic conditions. These included hearing impairment, visual impairment, neurocognitive impairment, a seizure disorder, hypopituitarism, cardiomyopathy, fatigue, anxiety, depression, and social isolation.

“I lost my hearing because of the operation and stuff. And just recently I think two, two years or four years ago I lost my hearing again. I see a doctor at X [hospital] but he said there’s no point for a hearing aid because like I always tell him I want the tiny ones… But he’s like you can’t because the hole is so big it could fall in [to the ear]. I can hear a little, I can hear a bit but not that well. But there’s no point, they said like, he said that maybe operate but he doesn’t want me to go through that procedure right now. I don’t want to go through surgery again… I have to wear the big ones [hearing aids] and I can’t because it will hurt here [ear] because with the glasses it, it bugs me.” [31-year-old, brain tumor survivor]

The survivors felt lost when they were expected to self-manage care and thus recognized that they were unable to manage independently. Comprehending medical information was an ongoing struggle. The survivor’s insight into their neurocognitive impairment coupled with frustrations with medical encounters and ineffective treatment resulted in poor self-confidence and limited perceived agency. Although they appreciated the unwavering parental support and assistance, the survivors resented this reliance because of their desire for independence. Yet, they worried about their future and could not imagine a life without their parents.

“My mum and my dad are my big supporters and I, I’d be, I tell my mum if, if we have to die I want us to go together or me first.” [31-year-old, brain tumor survivor]

In this narrative, the survivors were consumed with their everyday struggles and could not envision their life improving and thus expressed fear of the future.

## Discussion

The CCS in this study had not expected the medical and psychological late effects of their cancer treatment that arose over time and that often remained unresolved. CCS shared five main narratives that represent distinct ways that they managed their health challenges, these being *trying to forget cancer*, *trusting the system to manage my follow-up care*, *being proactive about my health*, *stumbling from one problem to the next*, and *struggling to find my way.*

Research has previously documented CCS knowledge deficits, both in the understanding of their initial disease and treatment and in the resultant risks [23, 28]. The survivors in this study who were unaware of their health risks were dismayed when they developed medical issues; however, late effects were also unexpected among those who recalled being informed of their risks. Perhaps these findings are in part due to an optimistic bias in health risk perception, as is common during young adulthood. CCS’ health risk perceptions have been documented as largely inaccurate, and in most cases, survivors underestimate their risks [50]. These findings could also reflect the inherent complexity CCS face in fully comprehending their previous treatment and the potential unintended consequences and varying abilities and proclivities to process this information. The communication of health risks is extremely difficult, in part because patients often lack the health literacy needed to understand medical discussions and many have low numeracy skills [51]. Risk communication is further complicated when conclusive evidence for even establishing an individual’s specific risk is lacking, as in the case of late effect risks facing CCS. It is unknown how effective clinicians are in presenting and communicating late effect risk information to CCS. It is also unknown how CCS make use of health discussions and information, if at all. Evidence that some survivors ignore this information comes from a recent American study wherein only 46 % of CCS reported ever having received a treatment summary despite the fact that all study participants were provided with a survivorship care plan [50]. Moreover, the content of information desired and used by CCS appears to change by initial diagnosis, treatment, and current age [52], and methods other than written (audio, visual, or web-based) may be required to address individual needs [53]. Emerging research also highlights the importance of conveniently accessed information, allowing survivors to review information as their needs and questions arise [54]. Further evidence is needed to determine how best to communicate with CCS about their existing health challenges and future health risks.

A number of the survivors, especially those who shared the narratives of *being proactive about my health*, *stumbling from one problem to the next*, and *struggling to find my way*, had long-standing worries and fears about cancer-related repercussions, a finding consistent with previous research [37–40]. When considered within the monitor/blunter cognitive coping style model [55, 56], these individuals demonstrated characteristics of high monitors, that is, they were knowledgeable of their health problems, perceived their health risks to be high, and had negative future expectations [57]. They also adhered to medical recommendations and coped effectively in the face of relatively minor health challenges, routine screening, for example, but experienced negative emotions and ineffective coping in response to severe health challenges. In contrast, CCS who predominantly shared the narratives of *trying to forget cancer* and *trusting the system to manage my follow-up care* appeared to be low monitors, or ‘blunters,’ who avoided health-related information and experienced less psychological morbidity. At the extreme, those who tried to forget cancer denied health challenges and failed to engage in adherence with medical care [55]. While this would require further research, it provides potential direction for intervention work with long-term CCS, considering that psychoeducational coping style-matched interventions are more effective than mismatched interventions [58–60].

Parents of adult CCS often continue to monitor survivor’s health and follow-up appointments [61], and according to our research, survivors also variously involve their parents in the management of their cancer-related health. Our findings suggest that there is a dynamic tension between adult CCS’s desire for independence and their lingering need for parental assistance. The survivors commonly described a process of assuming responsibility for and learning how to take care of their health and navigate the healthcare system as independently as they could. Clinicians have recognized the importance of beginning the process of transition well before survivors are discharged from pediatric providers and families might also benefit from counseling about how best to empower survivors to, for example, seek out medical assistance, access psychosocial support, schedule appointments, fill prescriptions, and speak with adult HCPs. In the long term, some CCS might also benefit from counseling to assist them to cope with their feelings of being misunderstood, abandoned, and unsupported by their parents.

A subgroup of parents, themselves, are also likely in need of psychological support over the long term. The CCS in our study who limited discussions and downplayed their health to protect their parents from worry and distress potentially recognized their parents’ persistent psychological vulnerabilities. Cancer-related worry, distress, continuing uncertainty regarding relapse and late effects, personal strain, post-traumatic stress symptoms, and concern about their child’s ability to care for themselves persist for some parents well into survivorship [62, 63]. Coaching parents in coping with their child’s disabilities might reduce parental stress and empower their parenting in everyday life [64], simultaneously improving survivor outcomes.

This study builds on previous research reporting both positive and negative stories about HCPs [38, 41], by suggesting that these exchanges and the nature of the patient-provider relationship influence the ways in which CCS interact with health services. Positive interactions contributed to CCS feeling confident that they were receiving appropriate medical care and, ultimately, promoted adherence with late effect screening and treatment recommendations. While provider knowledge deficits are well recognized [24–27], this study offers evidence that HCP lack of understanding, empathy, and willingness to listen and investigate CCS concerns can be a source of frustration, undermine the provider-survivor relationship, and operate as a barrier to basic late effect prevention and management. This is particularly concerning considering the chronic nature of many late effects, which necessitates an ongoing collaborative relationship between CCS and providers. These findings suggest that although HCP education regarding late effects is essential, perhaps this ought to be augmented by service provision structures that motivate providers to take additional care to nurture a trusting relationship and convey respect for survivors’ concerns. Fostering positive relationships might prove to be vital for survivors who suffer multiple debilitating medical, emotional, and neurocognitive late effects, such as those who shared the narrative of *struggling to find my way*, an idea warranting further research.

The cumulative evidence indicates that tailoring a therapeutic intervention to the individual patient based on specific models or theories of health behavior, such as the Health Belief Model, the Transtheoretical Model (Stages of Change model), or the Theory of Reasoned Action, can enhance patient outcomes [65–67]. A comparison of the five narrative themes in this study to existing health behavior theory is warranted, as is the testing and refining of these narratives with larger samples representative of the CCS population. These narrative themes could then serve as a framework for developing formal tools to assess survivor archetypes that represent different ways that CCS manage their health and engage with health services, thereby assisting HCPs to tailor their care to the specific patient archetype in ways that optimize the effectiveness and efficiency of their interactions with survivors. That is, clinicians could assess the dominant narrative theme shared by individual survivors and subsequently tailor their approach to providing health information, discussing screening recommendations, communicating treatment options and outcomes, involving patients in decision-making, encouraging self-management of late effects, facilitating lifestyle behavior change, and organizing psychosocial support, for example. This could be akin to assessing a patient’s readiness to change their health behavior and then providing guidance, encouragement, and advice accordingly [65].

The study findings provide insight into the patient-centered health service needs of CCS. First, the unresolved psychological challenges experienced by a subgroup of CCS highlight the need to incorporate routine psychological screening into standard care [68] and to improve the accessibility and effectiveness of psychological support. Chronic health conditions and impaired physical performance have been associated with poor psychological functioning and quality of life among CCS [10]. Particularly worrisome in this research were the reports of ineffective psychological support experienced by survivors with complex chronic conditions that contributed to their feeling a loss of hope and eventual rejection of services. Interventions are needed that target the specific and chronic issues facing survivors, help them cope more effectively with the burden of illness [10], assist them to make meaning out of their experiences, but also identify when support is ineffective and ought to be revised. Second, and in line with previous research [18], some CCS in this study reported being lost-to-follow-up, not knowing where to obtain medical care, and experiencing difficulty connecting with the appropriate HCP. At the time this research was conducted, there was no formal, coordinated health service structure for the provision of CCS long-term follow-up in British Columbia, as is still a common reality worldwide. In the absence of such service structures, CCS will continue to encounter circuitous routes to obtaining required care at best and be completely overlooked and disconnected from services at worst. Third, our research indicates that CCS possess a range of desire and ability to self-manage their health. Health service interventions that provide CCS and HCPs with accessible information, through survivorship care plans or passports to care [13, 24, 69], for example, might possess the greatest potential if augmented by pragmatic support that assists survivors to enact their agency as best they can. The CCS in our study commonly wanted to be treated as autonomous individuals, capable of engaging in medical decision-making and self-care, even when they required assistance.

Several limitations should be noted. All study participants were recruited in one Canadian province and were currently receiving some form of medical follow-up related to their cancer, whether it be through a LTFU clinic, a general practitioner, or walk-in-clinics. Thus, caution must be exercised when determining the relevance to other settings, especially with different service delivery structures, or CCS who may or may not have access to dissimilar follow-up services. Furthermore, the study participants likely represent CCS at higher risk for late effects considering that the majority had received radiation therapy and had subsequent medical and psychosocial late effects. In the USA, the use of radiation therapy (almost exclusively external beam) has declined from 1973 to 2008 in 7 of 10 pediatric cancer subtypes; acute lymphoblastic leukemia (57 to 11 %), non-Hodgkin lymphoma (57 to 15 %), retinoblastoma (30 to 2 %), brain cancer (70 to 39 %), bone cancer (41 to 21 %), Wilms’ tumor (75 to 53 %), and neuroblastoma (60 to 25 %) [70]. There were minimal changes in radiation therapy use for Hodgkin’s lymphoma, soft tissue cancer, and acute myeloid leukemia at 72, 40, and 11 %, respectively [70]. As such, it is expected that overall children treated for cancer today will suffer fewer radiation-related late effects. Also missing from this research are the experiences of survivors with minimal cancer-related health challenges or future risks who are not currently using health services. However, the perspectives of survivors who are severely neurocognitively and functionally disabled are underrepresented in this research.

## Conclusion

Considerable evidence now describes the late effects that often emerge long after children are cured of their original cancer. This study complements the existing literature by describing various ways that survivors themselves manage these health challenges and by providing patient-perspectives that draw attention to diverse ways that survivors enact their agency. Future prospective research that describes how different models of survivorship care shape survivors interplay with health services is currently needed, particularly considering the many different programs under development for this population. Research is also warranted that illuminates how educational, psychosocial, and health service interventions influence survivors’ experiences so that health outcomes can then be optimized and the burden of late effects minimized.
